# Interaction between *Cymbidium aloifolium* and *Apis cerana*: Incidence of an outlier in modular pollination network of oil flowers

**DOI:** 10.1002/ece3.8697

**Published:** 2022-03-14

**Authors:** Arjun Adit, Monika Koul, Ashish Kumar Choudhary, Rajesh Tandon

**Affiliations:** ^1^ 28742 Department of Botany University of Delhi Delhi India; ^2^ 28742 Botany Department Hans Raj College University of Delhi Delhi India

**Keywords:** elaiophores, floral constancy, floral lipids, morphometric matching, orchid, reproductive ecology

## Abstract

So far, oil‐rewarding flowers are known to be pollinated only by oil‐collecting bees, which gather and use lipids for larval feed and nest building. As honeybees do not have oil‐collecting appendages on their legs, they have not been associated with pollination of such flowers. In a predominantly *Apis* pollinated and food deceptive clade of wild Cymbidiums, we investigated the reproductive strategy of *Cymbidium aloifolium*, hitherto unknown for its floral oil reward. Our study demonstrates the requisites for establishment of mutualistic interaction between the oil flower and *Apis cerana indica*, a corbiculate bee. Success in pollination requires learning by honeybees to access the food reward, thereby displaying cognitive ability of the pollinator to access the customized reward. Morphometric matching between orchid flowers and the pollinator, and that between pollinia and stigmatic cavity also appear to be essential in the pollination success. Absence of pollinator competition and prolonged flower‐handling time are suggested to promote floral constancy. The present study highlights the need to explore the spectrum of pollination rewards pursued by honeybees, which may include unconventional composition of floral resources.

## Graphical abstract

*Cymbidium aloifolium* is a perennial, epiphytic orchid found in the tropical regions of South Asia. Its oil‐rewarding flowers are invariably pollinated by a non‐oil bee, *Apis cerana indica*. The study highlights the mechanism and traits that establish floral constancy and pollination mutualism. Photo: Arjun Adit

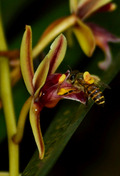

## 1 INTRODUCTION

The enormous variability and rate of speciation known among orchids are strongly attributed to their interaction with pollinators (Kiester et al., 1984; Paulus, 2019). However, information on the mechanistic basis for how such interactions are established among orchids is either limited or yet to be demonstrated. This is especially true for the species located in tropics, where Orchidaceae shows immense diversity (Corlett, 2004; Micheneau et al., 2009).

The extent of specialization in pollination systems is most pronounced in orchids. However, the degree of specialization may vary across the distribution range of plants (Pauw & Bond, 2011). It has been generally observed that pollinators tend to be more generalists than the plants (Schiestl & Schlüter, 2009). While specialist plants usually get attuned to one insect, the generalist pollinators may become opportunistically dependent on such plants due to gradual built up on a known resource (Minckley & Roulston, 2006). However, the pollinators develop a constancy for a flower only when they become effective in accessing the rewards or are able to recognize the refined cues (olfactory and/or visual) of attraction (Nilsson, 1992; Wells & Wells, 1986). Besides the requisites, drivers like (i) conducive phenology or the availability of flowers and suitable foragers (Marques et al., 2007), (ii) dimensional matching between flower and the pollinator (Chittka et al., 1999), and (iii) a non‐competitive pollinator environment (Grüter et al., 2011) are also considered crucial in establishing the constancy.

Besides nectar and pollen, flowers may offer lipids, oils, or waxes/resins as rewards (Simpson & Neff, 1981). These specialized rewards have been reported in the members of Calceolariaceae, Cucurbitaceae, Fabaceae, Gesneriaceae, Iridaceae, Krameriaceae, Malpighiaceae, Melastomaceae, Orchidaceae, Plantaginaceae, Primulaceae, Scrophulriaceae, Solanaceae, and Stilbaceae (Alves‐dos‐Santos et al., 2007; Buchmann, 1987; Machado, 2004; Rasmussen & Olesen, 2000; Simpson & Neff, 1981; Vogel, 1969). The flowers in some of these families have specialized epidermal secretory structures termed elaiophores, which produce non‐volatile oils as pollination reward. Occurrence of the elaiophores among angiosperms is a polyphyletic feature, with oil flowers evolving at least 28 times and being lost 36–40 times on independent occasions (Renner & Schaefer, 2010). On the basis of their structure, two distinct types of elaiophores have been categorized, *viz*., epithelial and trichomatous (papillate) types (Possobom & Machado, 2017).

Plants that produce oil from their floral organs are known to be exclusively pollinated by oil bees. These bees utilize the floral oils for building nest and reinforcement of the hives. However, the consumption of oil as a food source is thought to be doubtful (Possobom & Machado, 2017). Honeybees are not known to be involved in pollination of such flowers as they lack structural appendages required for oil collection; they are known to meet their dietary requirements of lipids (essential fatty acids) only by consuming pollen. It is now clear that honeybees can taste the fatty acids (Ruedenauer et al., 2021). However, it is still unclear if the floral oil‐producing plants could be pollinated by non‐oil bees, such as honeybees. Furthermore, in the absence of structural appendages, do bees collect lipids as an incentive, or feed on it?

The genus *Cymbidium* Sw. (Orchidaceae; Epidendroideae; Cymbidieae) includes 74 accepted species and has a distributional range across South and Southeast Asia as well as Oceania (POWO, 2020). Among the 15 wild *Cymbidium* species investigated so far, the nature of the reward is documented only for 7 of them; 6 of these are pollinated by deception (non‐rewarding) strategy. Among the tribe Cymbidieae, Cymbidiinae with six genera is one of the poorly investigated subtribes with respect to reproductive ecology. Here, we present our findings on the pollination strategy employed by *Cymbidium aloifolium* (L.) Sw., analysis of its floral reward, the prevailing breeding system, and provide evidence that its pollinator, *Apis cerana indica*, forages for the lipidic reward in a specialized pollination relationship at the study sites.

## 2 MATERIALS AND METHODS

### 2.1 Study species and populations investigated

*Cymbidium aloifolium*, commonly known as “Aloe‐leafed Cymbidium,” is a sympodial orchid native to South and Southeast Asia (POWO, 2020). This epiphytic orchid usually grows in dense clusters in forested as well as inhabited areas of the tropical region (Figure S1). Up to two racemes are borne in a plant, and each of them may bear ~25 flowers, which emit a mild fragrance. Tepals in a freshly opened flower are rigid and creamy white, with maroon‐red streaks in the middle. Peak flowering was noted during the second and third weeks of May.

The study was carried out in the natural populations in Tripura, India, during the peak flowering time between 2018 and 2020 (three flowering seasons): (i) Clouded Leopard National Park, Sepahijala (23.675°N, 91.320°E, 47 m), and (ii) Heritage Park, Agartala (23.856°N, 91.285°E, 13 m). A voucher specimen of the plant from the study area was submitted at the Delhi University Herbarium, University of Delhi, India (*DUH 14669*).

### 2.2 Floral biology

Flowers were monitored from bud stage to the formation of fruits (Figure S2). The most receptive stage of the stigma was ascertained using peroxidase test (Galen & Plowright, 1987). The labellum from the fresh flowers (*n* = 10 from each population) was subjected to various tests, including (i) neutral red to localize osmophores; (ii) Sudan Black B to detect elaiophores/ floral oils/lipids; (iii) Coomassie brilliant blue R (CBBR) to detect proteins; and (iv) Lugol's iodine (I_3_K) to detect starch (Shivanna & Tandon, 2014).

Labellum from freshly opened flowers were fixed in 2.5% glutaraldehyde (Sigma‐Aldrich), and subsequently stored in phosphate buffer (pH 7.2, 1N). For microtomy, capsules were prepared by embedding portions of labellum in 2‐hydroxyethyl methacrylate‐based resin monomer, following Feder and O'Brien (1968). The sections (4 and 5 µm) were obtained using a rotary microtome (AO Spencer, Model 820, USA). These were stained with toluidine blue O (TBO; Sigma‐Aldrich) for studying the structural details, and with Sudan Black B (Sigma‐Aldrich), auramine O’ (Sigma‐Aldrich), and neutral red (Sigma‐Aldrich), to localize the secretory tissue. Photomicrography was done using a bright‐field microscope (Zeiss Primo Star, Gottingen, Germany) mounted with a digital camera (Canon PowerShot G10, Tokyo, Japan). For scanning electron microscopy (SEM), fixed labellum samples were passed through an ascending dehydration series using cold acetone, and subsequently critical point dried (E‐3000, Quorum Technologies, UK). Post‐sputter coating with gold–palladium (JFC‐1600 Autofine coater, Jeol, Japan), the material was viewed under a scanning electron microscope (JEOL JSM‐6610LV, Japan).

### 2.3 Breeding system analysis

Breeding system was established by conducting four pollination treatments over the study period. For this, the freshly opened flowers were randomly selected in the populations; the treatments included: (i) Apomixis: pollinia were removed and flowers were bagged without pollination (*n* = 524 flowers in total; *n* = 20 individuals per year); (ii) Spontaneous autogamy: flowers were bagged without causing any disturbance (*n* = 513 flowers in total; *n* = 20 individuals per year); (iii) Geitonogamy: pollinia were manually deposited in the stigmatic cavity of a flower in the same inflorescence (*n* = 587 flowers in total; *n* = 20 individuals per year); and (iv) Xenogamy: pollinia for pollination were sourced from a different plant (*n* = 576 flowers in total; *n* = 20 individuals per year). Furthermore, some undisturbed flowers (*n* = 592 flowers in total; *n* = 20 individuals per year) were tagged and monitored for natural fruit‐set. Bagging was done using paper bags (7 × 8 cm) and removed after 24 h of the treatment. The values were expressed as percentage fruit‐set.

### 2.4 Pollination ecology

In order to investigate the reliance of the orchid species on pollinators, initially the observations were spread across the day (4:30 a.m. to 11:30 a.m.; 2:00 p.m. to 6:30 p.m.) as well as the nighttime (7:00 p.m. to 9:30 p.m.). After ascertaining that the pollinator activity is confined to diurnal hours, the observations were made accordingly. For recording the time of visitation, foraging behavior, and flower‐handling time, the plants were observed between 5:00 a.m. and 10:00 a.m. in the forenoon (total observation period = 225 h); observations were taken on a previously identified flowering patch (*n* = 50 individuals in each population) during peak flowering over three seasons (2018–2020).

The foraging frequency was ascertained by observing the number of flowers visited by a single bee in each bout/flight (*n* = 6 bouts per population, each season). Flower‐handling time of the pollinator was documented using a digital stopwatch (*n* = 10 flowers per population, in each season). The pollinating insects were collected using a net, and subsequently immobilized using ethyl acetate and stored in a plastic box lined with cotton (Dafni, 1992). These were later identified at the Entomology division of the Indian Agricultural Research Institute, New Delhi.

In *C*. *aloifolium*, the legitimate entry of the pollinator in the flower is located at the front (through the opening between the column and labellum); the reward exuding tissue is located in the mentum region of the labellum. The dimensions (width and height) of the opening (*n* = 100 flowers; 20 individuals) and that of the pollinator (*n* = 20) were measured with the help of a digital Vernier caliper (Insize, Model 1112‐150, India) to establish morphological matching between the two. While intertegular distance was measured for bee width; distance between femur and top of thorax was used to represent bee height. Morphometric details of the pollinia (*n* = 100 pollinia; 20 individuals), anther cap (*n* = 100 anther caps; 20 individuals), and the stigmatic cavity (*n* = 100 stigmatic cavities; 20 individuals) were also noted down. After each independent floral visit to a virgin flower, pollinators (*n* = 20 occasions) were captured and verified to see if reward was collected anywhere on the insect's body. Subsequently, labellum of the visited flowers (*n* = 40) was also investigated to validate foraging, and to see if the reward was removed.

### 2.5 Analysis of reward

Histochemical localization of proteins (CBBR), carbohydrates (periodic acid–Schiff's reagent), and lipids (Sudan Black B) in the resin‐embedded sections of mentum region was carried out to ascertain the chemical nature of the reward. As the histochemical tests of the exudate from the mentum responded only for floral lipids, we further carried out GC‐MS (gas chromatography coupled with mass spectrometry) analysis to find out the chemical composition of the exudate. For this, labellum of fresh and unpollinated flowers (*n* = 100; pooled from both the populations) were used for collecting the exudate by using sterile micro‐capillary tubes; the capillaries were immersed in vials containing ethyl‐acetate (Qualigens, 98%) (Reis et al., 2000). Fatty acid methyl esters (FAMEs) analysis was carried out as per the method described by Roughan and Nishida (1990) with modification. FAMEs were prepared by alkaline method using sodium methoxide. The samples were transferred into a conical glass tube and dried with Nitrogen gas. One milliliter sodium methoxide (Sigma‐Aldrich, 95%) and 15 µl toluene (Sigma‐Aldrich, 99.8%) were added to the samples and glass tubes were capped with Teflon‐coated screw caps. Samples were vortexed and kept at room temperature for 15 min and subsequently incubated at 37°C for 20 min. Post‐incubation, 100 μl glacial acetic acid (Qualigens, 99.5%) followed by 2 ml hexane (Sigma‐Aldrich, GC‐grade, ≥99%) was added and briefly vortexed. Samples were then centrifuged at  295 × *g* for 2 min and upper organic phase was transferred to a new glass tube. Samples were dried using nitrogen gas and dissolved in 50 μl hexane. From the prepared sample, 1 µl was subjected to GC‐MS (5977A MSD coupled with 7890B GC series equipped with a 30 m × 0.25 mm × 0.25 mm DB‐wax capillary column, Agilent Technologies) with a program described by Choudhary et al. (2017) (see Supplementary Information). The experiment was repeated and readings were noted for the replicates (*n* = 3). Three samples were taken from the same collection; and subsequently run on through the column, which was cleared off residue from the previous round. Mean of the triplicates were used as percent abundance of each fatty acid.

### 2.6 Plant–pollinator interaction among the wild Cymbidiums

Phylogenetic analysis vis‐à‐vis study of pollination interactions is important to understand many aspects of orchids, as they have adapted with finely tuned relations governing their fecundity and survival. For phylogenetic analysis, ITS 1–ITS 2 region sequences of *Cymbidium* spp. (Table S1) were retrieved in FASTA format from the NCBI (https://www.ncbi.nlm.nih.gov/) and aligned using MAFFT v.7.0 (https://mafft.cbrc.jp/alignment/server/). Maximum likelihood analysis with default parameters was performed using RAxML–HPC Blackbox tool in CIPRES science gateway v.3.3 (http://www.phylo.org/). Phylogenetic tree of the pollinator taxa was adapted from Cameron and Mardulyn (2001). Bipartite network was imported as binary data (presence and absence) in excel file based on information gathered from the literature (Table S2). The tanglegram and modularity were visualized using *ape* (Paradis & Schliep, 2019) and *bipartite* (Dormann et al., 2008) packages in R software (R core team, 2020), respectively.

## 3 RESULTS

### 3.1 Floral biology

Flowers of *C*. *aloifolium* are long‐lived and remain fresh (up to 20 days) until pollination is achieved. The unpollinated flowers are presented at right angle to inflorescence axis (Figure S3). Post‐pollination, the flowers droop down, petals turn yellow, and the ovary gradually begins to swell. One day after pollination, the swelling in the column gradually covers the stigmatic cavity and makes it inaccessible. Five days after pollination, the column begins to shrink, and persists in the mature fruits (Figure S2).

**FIGURE 1:**
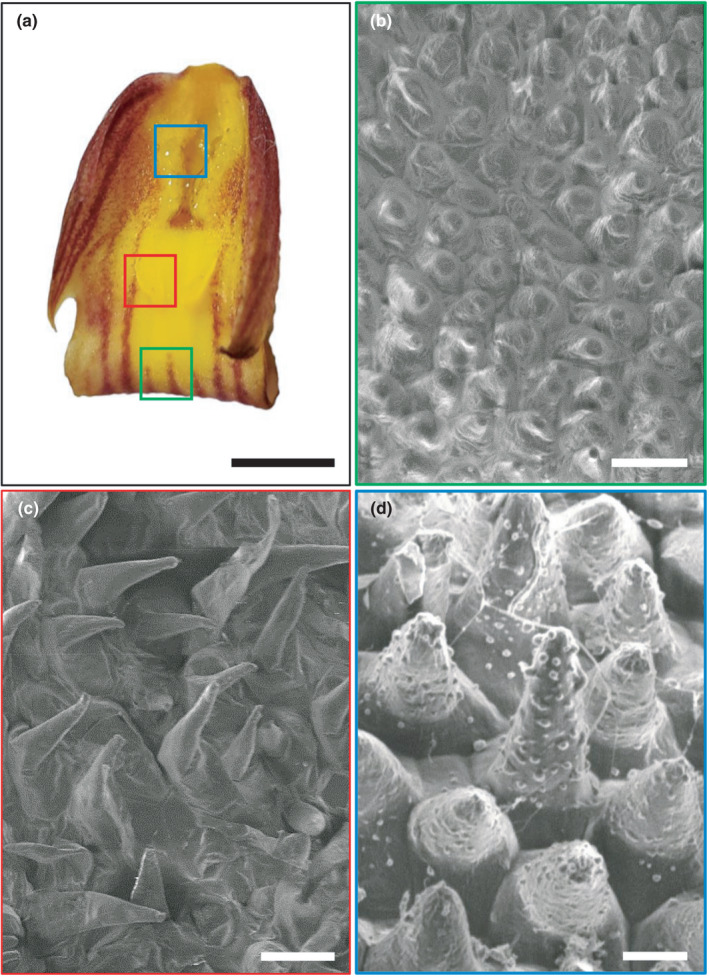
Micromorphology of a *C*. *aloifolium* labellum. (a) Whole mount of a fresh lip showing functionally important regions, landing platform (green box, b); site of volatile secretion (red box, c); and site of production and collection of floral lipids (blue box, d). (b) Conical cells on the landing platform. (c) Trichomatous osmophores on ornamentations of the lip. (d) Papillate elaiophores laden with lipid residue. Scale bar: a = 3 mm; b, c = 50 µm; d = 10 µm

**FIGURE 2:**
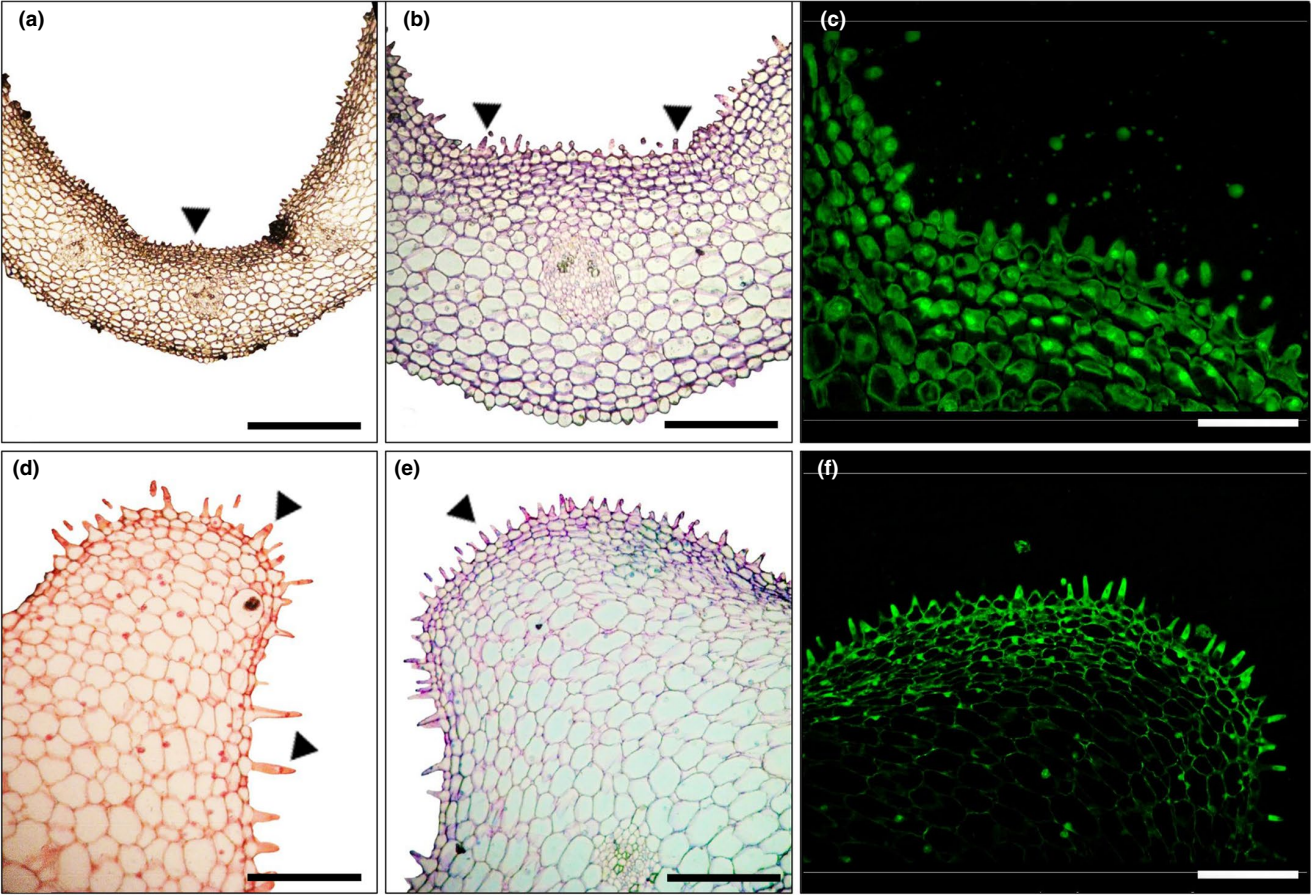
Resin sections of secretory regions on the labellum of *Cymbidium aloifolium*. (a–c), Elaiophore region stained with Sudan Black B, showing the dermal layers of cells (arrowhead) with accumulated lipids (a); with toluidine blue O showing unicellular and bicellular papillae (arrowhead) (b), and with auramine O’ (c). (d–f), Portion of the protuberance stained with neutral red to localize osmophores (arrowhead) (d); with toluidine blue O showing unicellular trichomes (arrowhead) (e), and with auramine O’ (f). Scale bar: a = 1 mm; b, d = 0.5 mm; c = 50 µm; e, f = 100 µm

Ultrastructural study revealed that the labellum surface is covered with trichomes, with three distinct regions—the distal broad region with conical epidermal cells, the middle portion with protuberances bearing osmophores, and the mentum which exudes shiny, viscous droplets of the reward (Figure 1; Figures S4 and S5). Resin‐embedded sections of the mentum region (positive for lipids) bear elaiophores, while the protruded region (positive for volatiles) have unicellular trichomes (Figure 2).

### 3.2 Breeding system analysis

Flowers bagged to ascertain spontaneous autogamy as well as apomixis failed to set fruits. However, the remaining treatments resulted in 100% fruit‐set, thereby establishing that in nature, *C*. *aloifolium* is a self‐compatible species. Since treatment for facilitated autogamy resulted in fruit‐set, selfing in this orchid is possible only with the aid of a pollinator. Fruit‐set through open pollination (control) was 37.6%.

### 3.3 Pollination ecology

**FIGURE 3:**
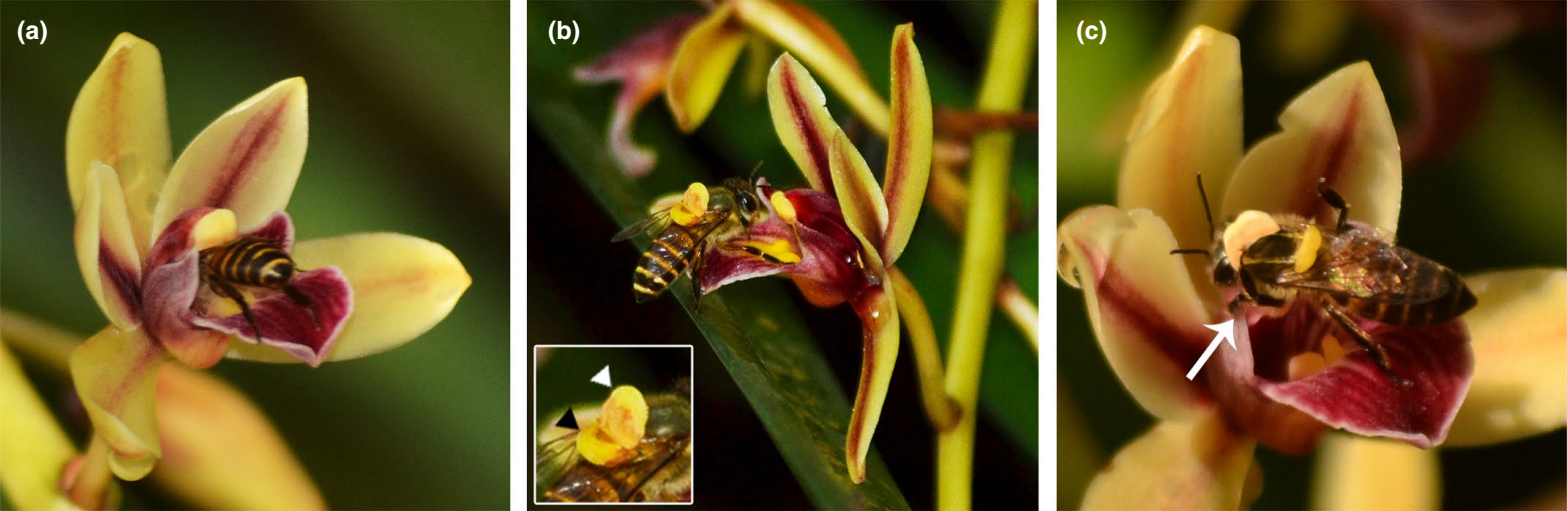
Pollination events in *Cymbidium aloifolium*. (a) An *Apis cerana indica* bee foraging the flower for rewards. (b) Nototribic collection of pollinia on the thoracic region; inset: with an anther cap (white arrowhead) and the pollinia (black arrowhead). (c) Bee actively separating the anther cap from the pollinium using its middle leg (long arrow)

**FIGURE 4:**
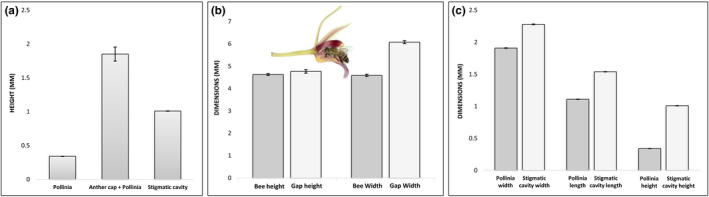
Morphometric matching of functional features during pollination. (a) Mean values of pollinia height, anther cap +pollinia height, and the stigmatic cavity height show that anther cap is a hindrance in lodging of pollinia into the stigmatic cavity. (b) Mean values of legitimate flower entry (white) and the corresponding dimensions of *Apis cerana indica* (grey) show dimensional matching between the two; inset: longitudinal section of flower with a captured *A*. *c*. *indica* placed on the labellum to depict its reach inside the flower. (c) Dimensions of pollinia (grey) and stigmatic cavity (white) show that only one pollinium can be accommodated in the cavity

Flowers of *C*. *aloifolium* were pollinated by *Apis cerana indica* in both the populations during the study period (Figure 3). The pollinating bees dimensionally matched with the opening of legitimate route of the flower (Figure 4). The protuberance of the labellum elevates the thorax to enable contact between bee and flower's column. The reward was invariably presented at the time of anthesis (early morning; 5:00 a.m.). Pollinator activity started by 6:00 a.m. and reached its peak between 7:00 a.m. and 8:00 a.m., subsequently ceasing by 9:00 a.m. As the reward is produced in trace amount, the bees visited many flowers in a single bout (32.71 ± 6.75 flowers per bout; *n* = 36 bouts). On an average a bee spent 13.14 ± 3.80 s (*n* = 60 observations) on a rewarding flower.

Active foraging of flowers led to deposition of pollinaria on the thoracic region of the bee (nototribic collection); pollination occurred after the bee visited another fresh flower (Video S1). The anther cap on the loaded pollinaria physically hampers the access of bees to reach the deep‐seated reward site (Figure 4a). This happens for three or four times, until the bee finally learns to actively get rid of obstructing anther cap from its back and makes its way into the flower. The bee uses its middle legs to separate the anther cap from pollinia, and only then, it manages to successful entry into the flower (Figure 3c). As the retreating bee stands firm with extended hind legs on the labellum, its thorax aligns in a nearly horizontal position, pushing the bee scutellum against the rostellum (viscidium) of the flower. Only one pollinium could be accommodated in a stigmatic cavity (*n* = 100 open‐pollinated flowers; Figure 4c). Neither the pollinator nor labellum of the visited flowers showed any trace of exudate on the surface post‐floral visitation, suggesting that the honeybee consumes the reward present in the mentum.

### 3.4 Analysis of reward

**FIGURE 5:**
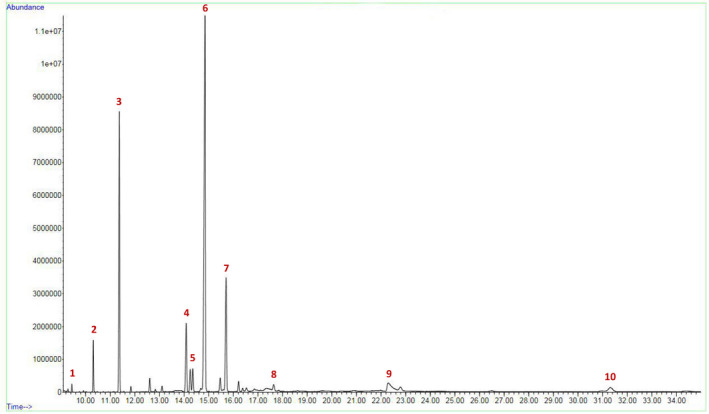
Profile of the labellar exudate of *C*. *aloifolium* obtained from GC‐MS. Peaks correspond to the following fatty acids: (1) myristic acid; (2) pentadecanoic acid; (3) palmitic acid; (4) stearic acid; (5) oleic acid; (6) linoleic acid; (7) α‐linolenic acid; (8) arachidic acid; (9) behenic acid; and (10) lignoceric acid

The analysis of the exudate revealed its predominant lipidic nature comprising a range of fatty acids (Figure 5; see Table S3 for abundance report). The exudate primarily contained linoleic acid (49.063%), palmitic acid (22.171%), α‐linolenic acid (12.570%), and stearic acid (7.136%). The other fatty acids were present in traces (each <4%).

### 3.5 Plant–pollinator interaction among the wild Cymbidiums

**FIGURE 6:**
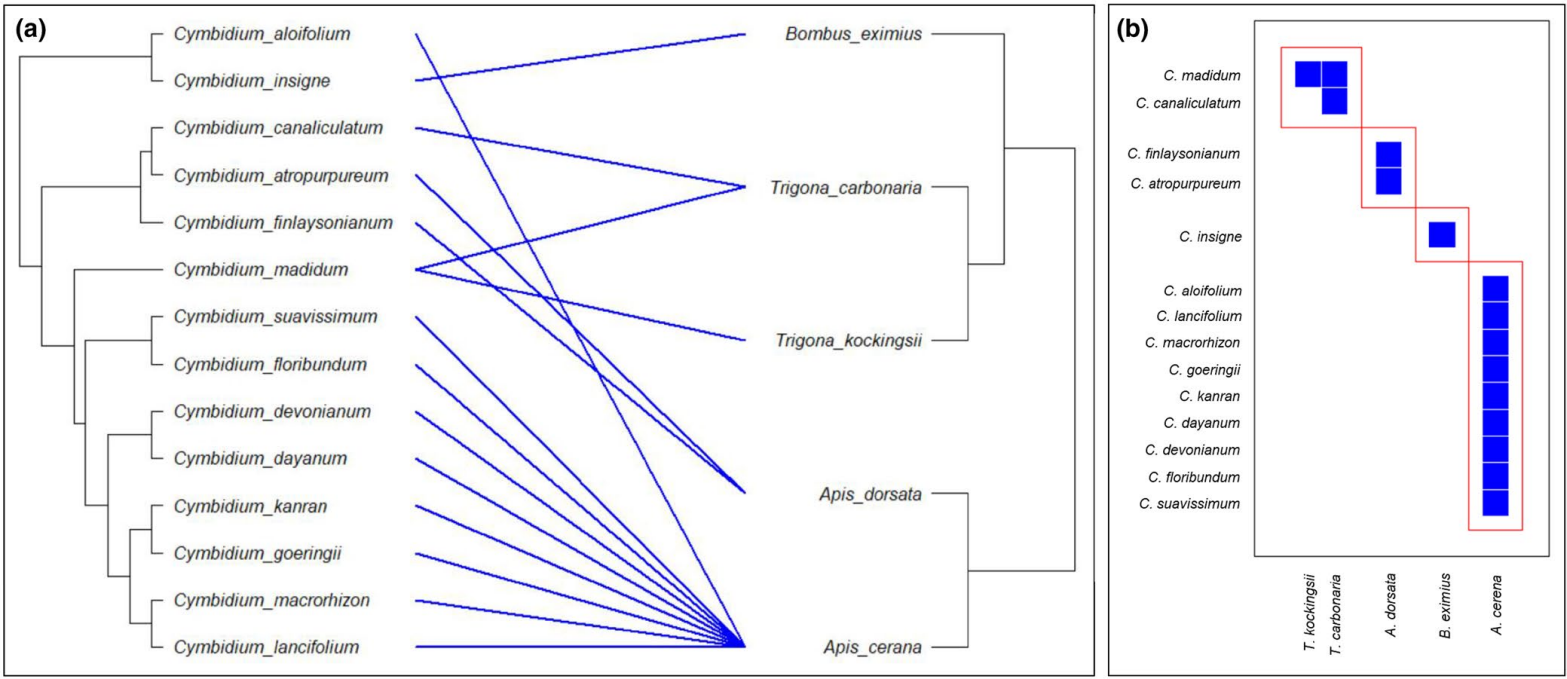
Interactome between wild *Cymbidium* spp. and their pollinators. (a) Tanglegram representing the bipartite relationship between the mutualistic partners. (b) High modularity between the two mutualistic partners is an implication of specialized pollination relationships

In general, the relationship between wild Cymbidiums and the Apidae pollinators indicates a highly modular network. Pollination in the clade seems to be specialized, with the majority of species interacting with *Apis* honeybees. The tanglegram does not give the impression of nested co‐evolution between the two partners; on the contrary, pollination in wild Cymbidiums seems to be driven by morphometric matching and floral constancy, as flowers with larger entry for mentum region (*C*. *insigne*, *C*. *atropurpureum*, and *C*. *finlaysonianum*) are pollinated by larger bees (*Bombus eximius* and *Apis dorsata*). Amid this bipartite network dominated by deception strategy, *C*. *aloifolium* is the only known taxon to achieve pollination success with a rewarding incentive (Figure 6).

## 4 DISCUSSION

Flowers with intricate architecture serve as filters to select a suitable pollinator, for maximizing pollination efficiency and maintaining the species identity (Barman et al., 2018; Laverty, 1994). Flowers of *C*. *aloifolium* have a showy labellum; it serves as the site of olfaction, landing platform, as well as production and presentation of the reward. The location and arrangement of supportive tissues of these functions are responsible for attraction of the pollination and assurance of pollination removal, and subsequent deposition in the stigmatic cavity. The landing platform supported by conical epidermal cells is located at the distal region, followed by osmophores in the middle and then the reward‐producing elaiophores at the base of column. This sequence of presentation of cue and reward not only necessitates the pollinator to forage with an effort to reach the core of flower but also ensures appropriate contact with essential organs. Significance of deep‐seated location of elaiophores in the flower seems to be crucial to achieve pollination success as the habituated pollinator is lured to it (Pansarin et al., 2009).

Our results regarding pollination‐induced stigmatic closure, changes in perianth, and swelling of column are in agreement with some of the investigated taxa belonging to tribe Cymbidieae (Clifford & Owens, 1988). Drooping of flowers to prevent subsequent pollinator visitation is an additional post‐pollination change observed in this plant. In *C*. *floribundum* Lindl., change in color from white to purple after pollination is observed in labellum (Sugahara et al., 2010). All these features are instrumental in dissuading unnecessary visitations on pollinated flowers.

As found in other *Cymbidium* species (Cheng et al., 2007; Matsuda & Sugiura, 2019; Suetsugu, 2015; Yu et al., 2008), the outcome of pollination treatments established that *C*. *aloifolium* is self‐compatible. Complete failure of fruit‐set through spontaneous autogamy, and a significantly lower fruit‐set in open‐pollinated flowers (control), indicate essential reliance on a suitable pollinator. Yet, natural fruit‐set of nearly 38% in *C*. *aloifolium* is higher than the mean fruit‐set observed among tropical orchids (~17%, Tremblay et al., 2005), which indicates high pollination efficiency in nature. In our work, the reason for reduced natural fruit‐set in comparison to the pollination treatments could be attributed to peak cyclonic activity in the Bay of Bengal (2018: Cyclone Daye; 2019: Cyclone Fani; 2020: Cyclone Amphan), which coincided with the flowering season. During strong winds and heavy rainfall, the pollinator did not visit the flowers. There was also loss of both pollinated and unpollinated flowers in strong winds. In general, pollinator activity gets disrupted under inclement weather conditions, and this has been recorded in a few hawkmoth‐pollinated Aerangoid orchids as well (Martins & Johnson, 2007).

Flowers of *C*. *aloifolium* present two key morphological adaptations for pollination success—first, ensures selection of a legitimate pollinator, and the second, conspecificity of pollinia. The morphometric matching of pollinia and stigmatic cavity which ensures conspecificity has also been noted in few Mediterranean orchid species (Lussu et al., 2019). However, perfect morphometric matching, especially between the legitimate opening and pollinator also appeared to be an impediment for the species, as the accessibility to the reward is not smooth, and only facilitated the entry after labellum is pushed down as a result of bee landing. Complete access is ensured only after the bee learns to remove further hindrance imposed by the protruding anther cap. The latter is actively removed by the bee after some attempts, unlike other orchids where the anther cap falls off on its own after some time (Dressler, 1981). Such foraging behavior is possible only if the bee learns to do so. The abundance of floral resources in a non‐competitive pollinator environment seems to assure a prolonged flower handling and helps the pollinator to learn the manner to seek the reward (Lichtenberg et al., 2020; Wcislo & Tierney, 2009). Such information is often stored as long‐term memory of honeybees, which aids in locating and identifying the flowers in constant relationships and is not erased by competing information (Chittka et al., 1999). This behavior among *Apis* bees is likely involved in permitting constancy and fidelity with suitable rewarding flowers in conducive environments.

The lipidic floral secretion is usually exuded in traces, and yet it seems sufficient in establishing mutualism. Incidentally, the flowers adapted to produce oils also require a specialized plant–pollinator interaction (Pansarin et al., 2020). Being a product of an energy‐demanding biosynthetic pathway, their amount in the flower is small and thus permits fewer floral visits than in those seen in carbohydrate‐rich nectar‐rewarding flowers (Heinrich, 1975; Simpson & Neff, 1981). The main source of lipids in honeybees’ diet is pollen, which are rich in linoleic acid, palmitic acid, linolenic acid, oleic acid, myristic acid, and stearic acid (Lepage & Boch, 1968; Manning, 2001). The composition of floral secretion in *C*. *aloifolium* is in coherence with these fatty acids, which are otherwise known to be involved in synthesis of intracellular reserves and membrane structure of bees (Graham, 1992). Fatty acids in general have anti‐bacterial and antifungal properties which help in keeping the honeybee disease free (Feldlaufer et al., 1993; Pandey et al., 1983).

Oil‐rewarding flowering plants have been linked with oil‐collecting bees belonging to Melittidae, Centridini, Ctenoplectrini, Tapinotaspidini, and Tetrapediini. There is, however, no evidence that these bees consume oils (Michener, 2007; Possobom & Machado, 2017). As the Apini members bear corbiculae instead of combs, hairs, or pads (as in the case of oil‐collecting bees), necessary for oil collection, their role in pollination is long believed to be doubtful (Martins et al., 2014; Medved et al., 2014; Neff & Simpson, 2017; Vogel, 1974). However, there have been no study to prove the involvement of non‐oil‐collecting bee(s) in the pollination success of oil‐rewarding plant. Furthermore, it is known that honeybees can perceive and taste fatty acids (lipids) in pollen grains (Ruedenauer et al., 2021), but evidence of bees directly foraging on a lipid concoction has never been presented. Our study establishes the involvement of *A*. *c*. *indica* in pollination of oil‐secreting flowers. Through the study, it is also evident that the honeybee is consuming the lipid‐rich secretion, and not collecting it as in case of oil bees. As the pollinia were never damaged by the bees, it indicates that pollen grains are not an incentive in *C*. *aloifolium*. Although few individuals of *Trigona* sp., a known oil‐collecting bee, were observed in the study area, they never visited the flowers. This may be due to presence of deep‐seated lipid secretions in *C*. *aloifolium*, as opposed to their usual hosts (conventional oil offering plant species), which have easily accessible open flowers. Besides, these stingless bees mismatch with the flower entry in *C*. *aloifolium*.

*Cymbidium* genus is exclusively pollinated by the members of Apidae family (Table S2). Despite such close affinity between the two, it may be too early to describe if this is a common feature in the subtribe or the tribe, for the want of investigation in other related taxa. However, it is evident from the dataset that *Apis cerena* and *Bombus eximius* associate with deceptive *Cymbidium* species. Renner and Schaefer (2010) hypothesized that Cymbidieae tribe has undergone nine independent origins of oil‐offering flowers. However, their inference does not account for the genus *Cymbidium* (subtribe Cymbidiinae). With oil‐rewarding flowers of *C*. *lowianum* (Davies et al., 2006) and fatty‐acid‐rich reward of *C*. *aloifolium* (present study), the number of independent origins in this tribe now becomes 11. This is consistent with the fact that acquisition of oil presentation as reward in Orchidaceae has multiple independent origins, as is the case in Iridaceae (Neff & Simpson, 2017). However, due to unresolved species complexes, and phylogeny in Orchidaceae, a precise number is uncertain (Chase et al., 2009).

Our study demonstrates the manner in which mutualistic interaction between *C*. *aloifolium* and *A*. *c*. *indica* is established. Morphometric matching between the plant and pollinator as well as that between the essential organs were crucial in pollination success. The pollinator exercises its cognitive abilities to get rid of the obstruction and reach the energy‐rich reward. Although produced in trace amount, the lipidic concoction offered by the habituated pollinator is sufficient to engender constancy between the two. Plant–pollinator interaction data on more *Cymbidium* species is needed to infer whether relationship between *Cymbidium*–Apidae is a result of nested co‐evolution.

## CONFLICT OF INTEREST

Authors declare no competing interests.

## AUTHOR CONTRIBUTIONS

**Arjun Adit:** Conceptualization (equal); Data curation (lead); Formal analysis (lead); Investigation (lead); Methodology (lead); Software (lead); Visualization (lead); Writing – original draft (lead); Writing – review & editing (equal). **Monika Koul:** Supervision (equal); Writing – review & editing (supporting). **Ashish Kumar Choudhary:** Methodology (supporting). **Rajesh Tandon:** Conceptualization (equal); Funding acquisition (lead); Project administration (lead); Resources (lead); Supervision (equal); Writing – review & editing (lead).

## Supporting information

Supplementary Material

Video S1

## Data Availability

All data generated during this study are included in the article (and its supplementary information files).
