# Supplementary material for: Interaction between Cymbidium aloifolium and Apis cerana: Incidence of an outlier in modular pollination network of oil flowers
Source: Ecol Evol. 2022 Mar 14;12(3):e8697. doi: 10.1002/ece3.8697 (PMC8928877; doi:10.1002/ece3.8697)
Supplement: Supplementary file 1 — Supplementary Material [file ECE3-12-e8697-s002.pdf]

SUPPLEMENTARY MATERIALS

Interaction between *Cymbidium aloifolium* and *Apis cerana*: incidence of an outlier in modular pollination network of oil-flowers

Arjun Adit[1], Monika Koul[2], Ashish Kumar Choudhary[1], Rajesh, Tandon[1*]

[1] Department of Botany, University of Delhi, Delhi, India

[2] Botany Department, Hans Raj College, University of Delhi, Delhi, India

FIGURE S1 A patch of *Cymbidium aloifolium*, an epiphytic orchid, in full bloom.

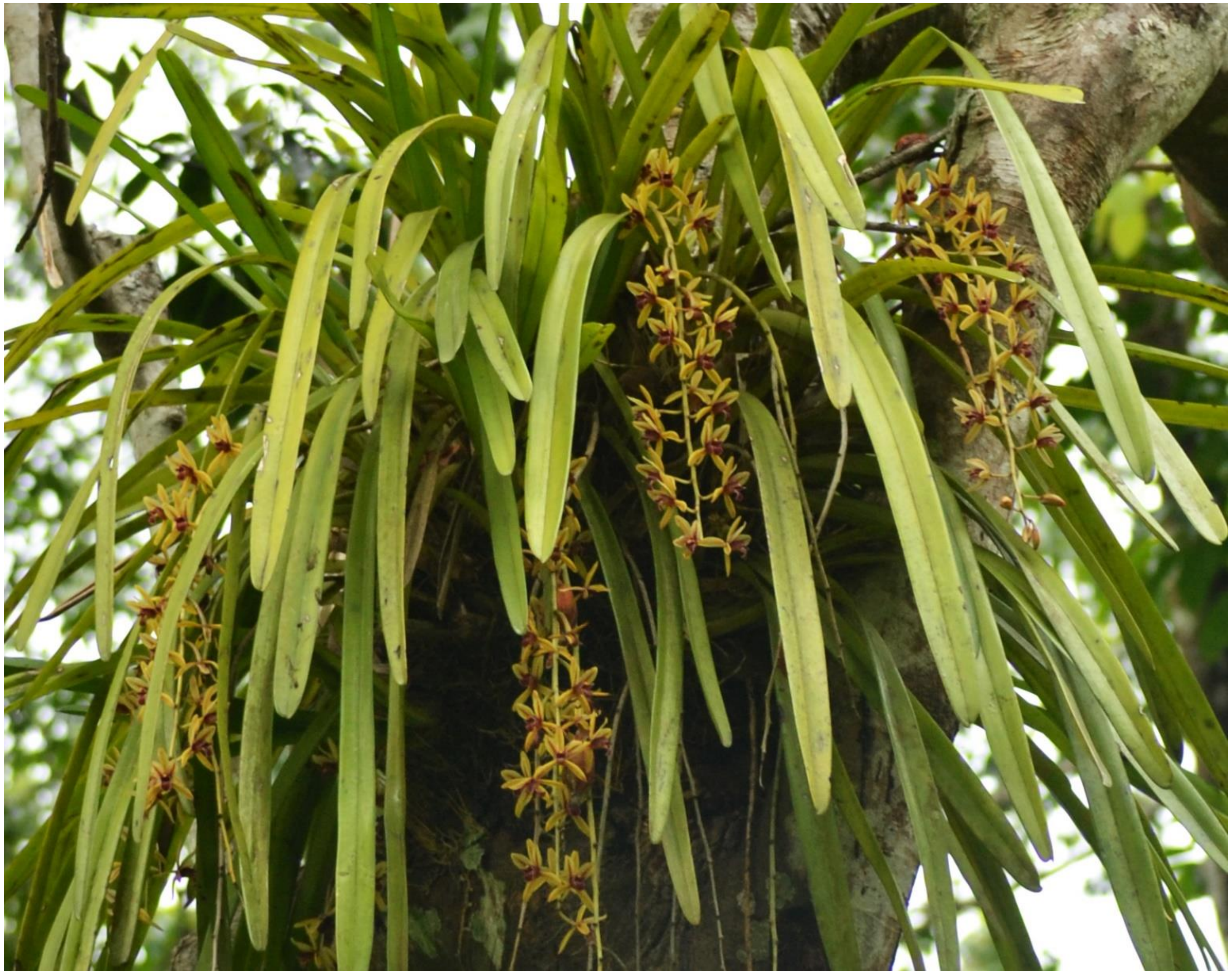

FIGURE S2 Floral stages of *C. aloifolium* in frontal (top) and apical (bottom) view. Abbreviations: DBA, day(s) before anthesis; DOA, day of anthesis; DAP, day(s) after pollination.

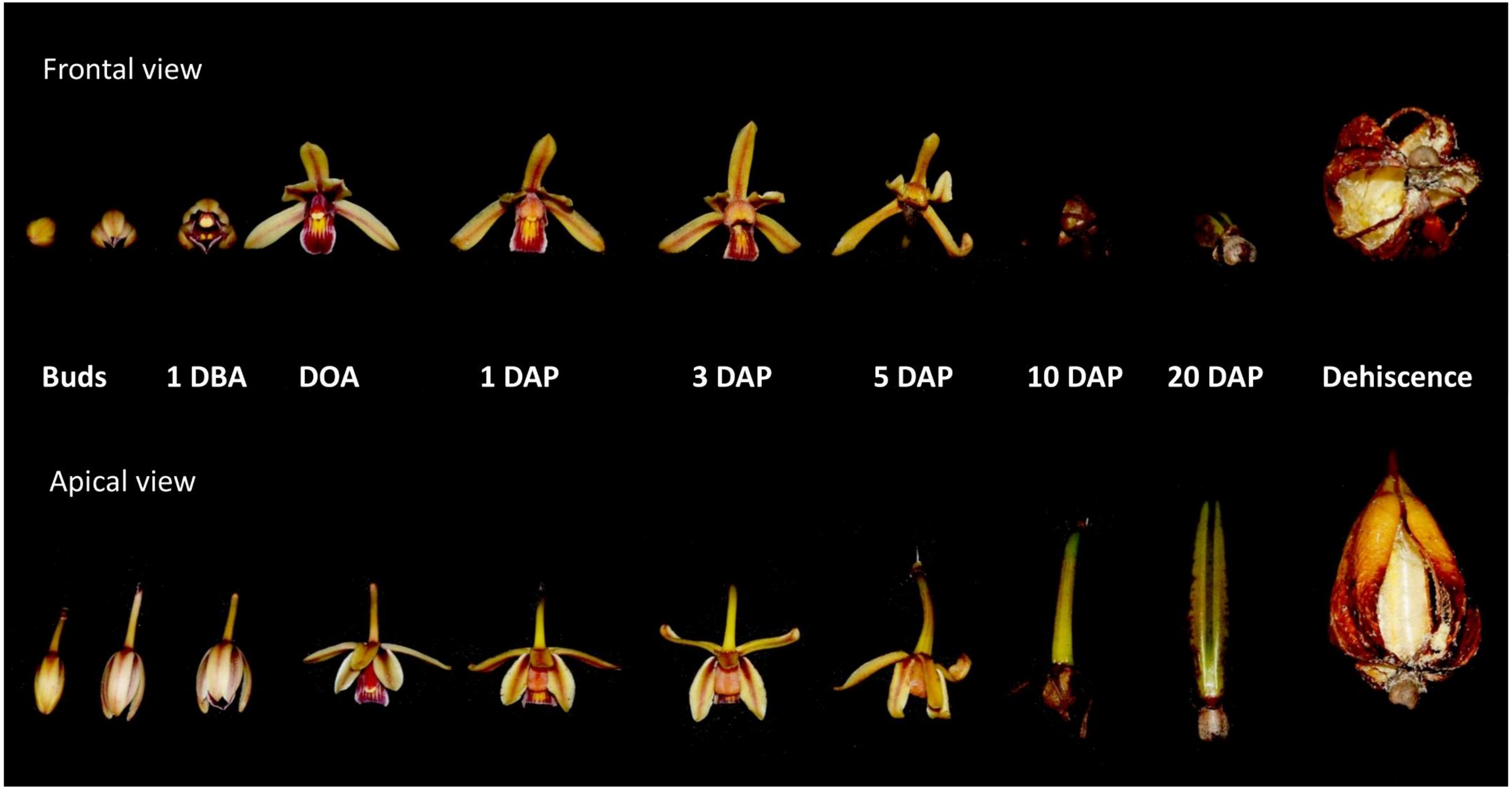

FIGURE S3 Post-pollination response in a flower of *C. aloifolium*. Pollinated flower droops down (white arrow) to deter further visitations from pollinator, while un-pollinated flower (black arrow) with intact pollinarium remain erect in the inflorescence.

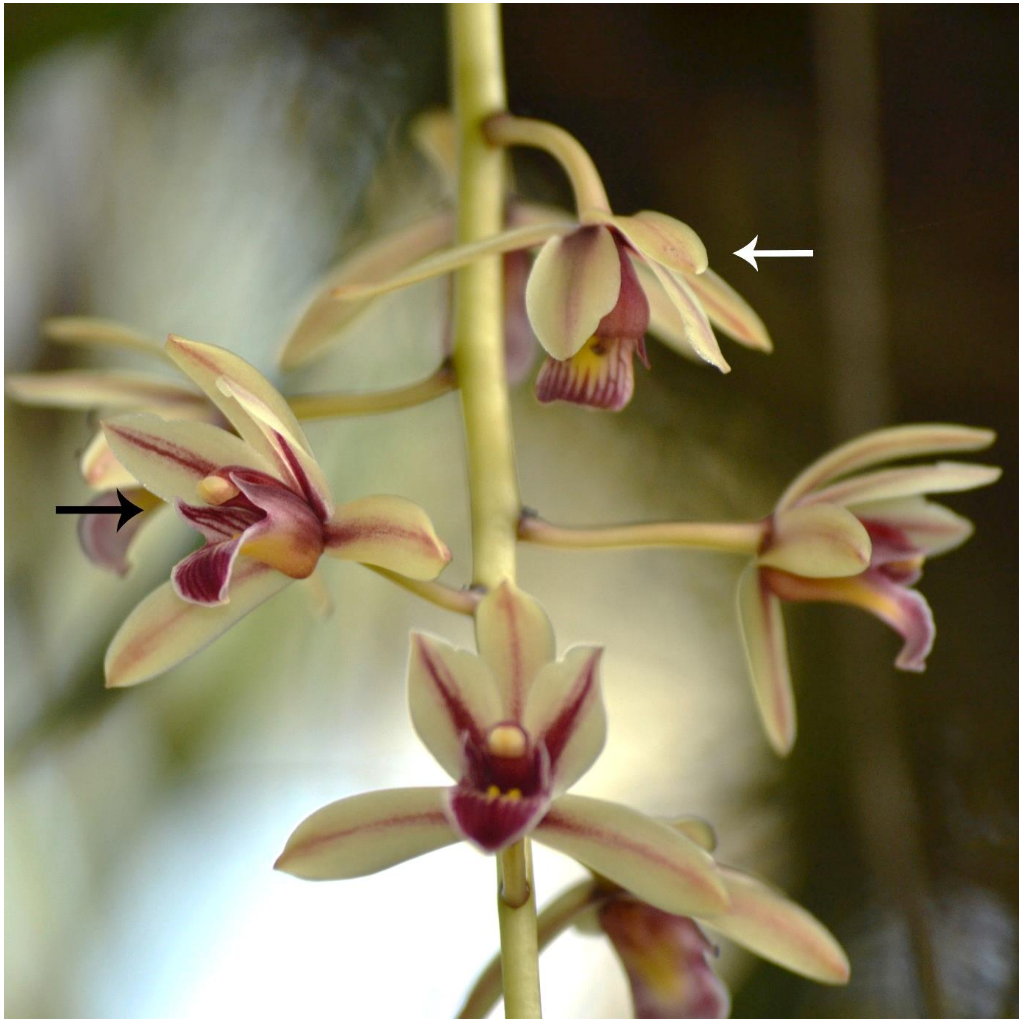

FIGURE S5 Scanning electron micrograph of the labellum (in apical view) reveals a trichome carpet all along the surface. Note the gradual change in density of trichomes from the osmophores region (black arrow heads) to the elaiophores region (white arrow head). Scale bar=1 mm.

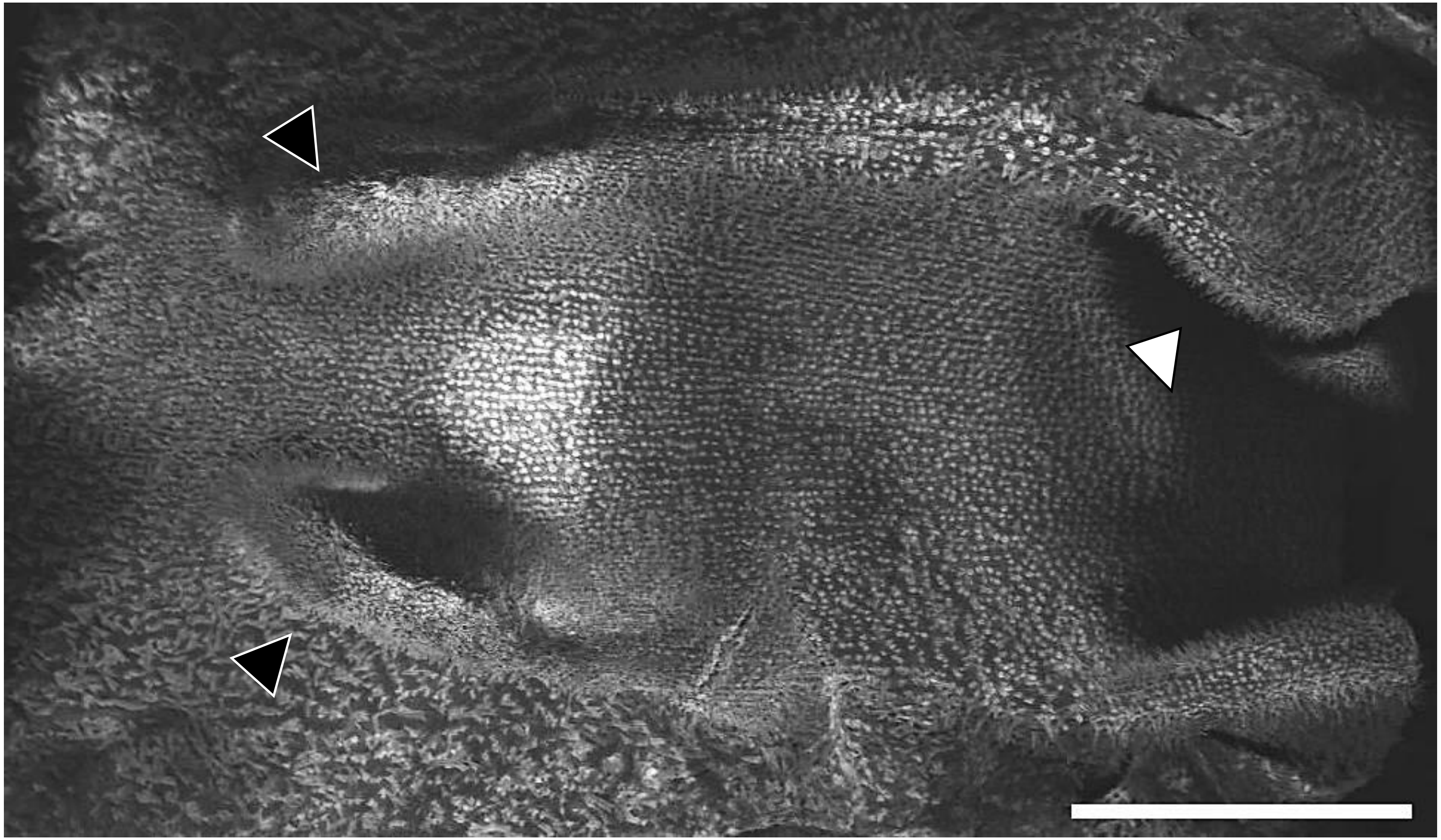

FIGURE S4 Labellum separately stained with various dyes to localise (a) lipids (Sudan black B); (b) proteins (Comassie brilliant blue R); (c) starch (Lugol's iodine); and (d) volatiles (Neutral red). The lipids were localised (red circle) in the mentum region while osmophores (white circle) on the protuberances in the middle region. Scale bar=6 mm.

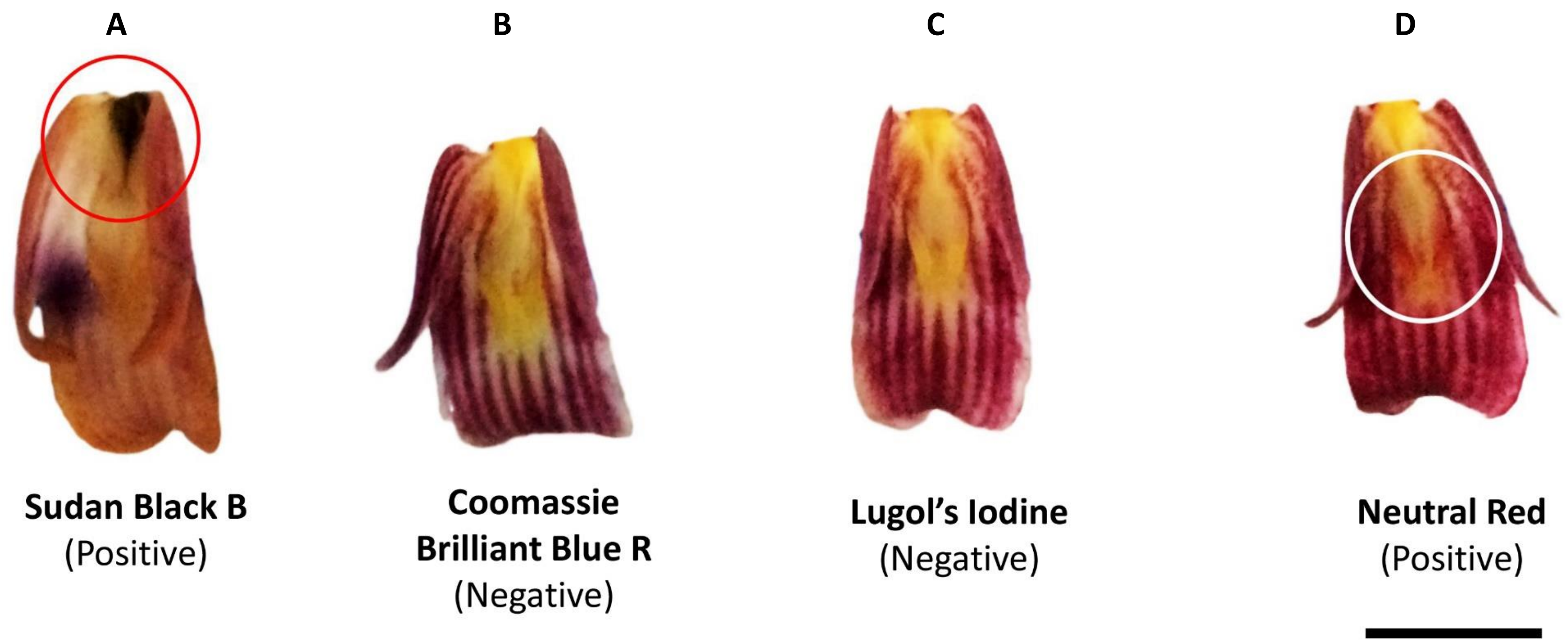

Details of programme used for GC-MS analysis

Gas chromatography-mass spectrometer (Agilent Technologies, 5977A MSD coupled with 7890B GC series) equipped with DB-wax column (30 m x 0.25 mm x 0.25 μm) was used for the analysis of fatty acid methyl esters (FAMEs). The carrier gas (Helium-99.9%) pressure was kept at 20.90 Psi at the inlet with a column flow rate of 1.8 ml/min and the GC oven temperature was maintained at 150 ^{o}C. The parameters of the column program were set as follows: an initial temperature of 50 ^{o}C for 3 min, then a ramping of temperature at the rate of 25 ^{o}C/ min up to 230 ^{o}C, and finally holding of temperature at 230 ^{o}C for 18 min. One μL of sample was injected with a split ratio of 20:1. Finally, the compound identification of the various FAMEs detected was identified by matching against the reference mass spectral database in the NIST library.

TABLE S1 List provides ITS accessions used in constructing a *Cymbidium* spp. phylogeny which is represented in FIGURE 6.

S. No.	Name	Accession no.
**1**	*C. aloifolium*	AJ300269
**2**	*C. atropurpureum*	AF470497
**3**	*C. canaliculatum*	MF861161
**4**	*C. dayanum*	AF284697
**5**	*C. devonianum*	JF729005
**6**	*C. finlaysonianum*	AF470514
**7**	*C. floribundum*	AF284698
**8**	*C. goeringii*	KF029638
**9**	*C. insigne*	AJ300270
**10**	*C. kanran*	AF284720
**11**	*C. lancifolium*	AF284724
**12**	*C. lowianum*	JF729007
**13**	*C. macrorhizon*	KT338679
**14**	*C. madidum*	AF470493
**15**	*C. suavissimum*	AF284700

TABLE S2 Pollination studies in the genus *Cymbidium*. (AS=Autonomous selfing; FS=Facilitated selfing; G=Geitonogamy; X=Xenogamy)

S. No.	Name	Pollinator	Incentive/ Cues	Breeding system	Pollination syndrome	Reference
1	*C. aloifolium* (L.) Sw.	*Apis cerena indica*	Lipids	FS/G/X	Reward pollination	Present study
2	*C. atropurpureum* (Lindl.) Rolfe	*Apis dorsata*	-	-	-	Chan *et al.*, 1994
3	*C. canaliculatum* R.Br.	*Trigona* sp.	-	-	-	Adams *et al.*, 1992
4	*C. dayanum* Rchb.f	*Apis cerena japonica*	-	FS/G/X	-	Matsuda & Sugiura, 2019
5	*C. devonianum* Paxton	*Apis cerena cerena*	-	-	-	Sugahara, 2006
6	*C. finlaysonianum*	*Apis dorsata*	-	-	-	Ong, 2010
7	*C. floribundum* Lindl.	*Apis cerena japonica*	Morphology and Fragrance	-	Food deception; Sexual Deception	Sasaki *et al.*, 1992; Sugahara *et al.*, 2010
8	*C. goeringii* (Rchb.f.) Rchb.f.	*Apis cerena cerena*	Fragrance	FS/G/X	Pseudopollen; Food deception	Yu *et al.*, 2008; Tsuji & Kato, 2010
9	*C. insigne* Rolfe	*Bombus eximius*	Morphology	FS/G/X	Food deception	Kjellson *et al.*, 1985
10	*C. kanran* Makino	*Apis cerena cerena*	Fragrance	FS/G/X	Food Deception	Tsuji & Kato, 2010
11	*C. lancifolium* Hook.	Autonomous selfing; *Apis cerena cerena*	Morphology	AS/FS/G/X	Food deception	Suetsugu, 2015
12	*C. lowianum* (Rchb.f.) Rchb.f.	-	Lipids	-	-	Davies *et al.*, 2006
13	*C. macrorhizon* Lindl.	Autonomous selfing; Apis cerena cerena	None	AS/FS/G/X	Food deception	Cheng *et al.*, 2007; Suetsugu, 2015
14	*C. madidum* Lindl.	*Trigona kockingsii* and *Trigona carbonaria*	-	-	-	Macpherson & Rupp, 1935; Smythe, 1970; Adams *et al.*, 1992
15	*C. suave* R.Br.	*Trigona kockingsii* and *Trigona carbonaria*	-	-	-	Adams *et al.*, 1992; Bartareau, 1993
16	*C. suavissimum* Sander ex C.H. Curtis	*Apis cerena cerena*	-	-	-	Sugahara, 2006

TABLE S3 Fatty acid composition of lipid secretion from *Cymbidium aloifolium* labellum.

S. No.	Fatty acid	% Abundance (Mean of Triplicates)
1	9, 12 - Octadecadienoic acid (Linoleic acid)	49.06 ± 1.45
2	Hexadecanoic acid (Palmitic acid)	22.17 ± 0.18
3	9, 12, 15 - Octadecatrienoic acid (α - Linolenic acid)	12.57 ± 0.29
4	Octadecanoic acid (Stearic acid)	7.13 ± 0.24
5	Pentadecanoic acid	3.15 ± 0.36
6	9- Octadecenoic acid (Oleic acid)	2.46 ± 0.35
7	Tetracosanoic acid (Lignoceric acid)	1.59 ± 0.35
8	Eicosanoic acid (Arachidic acid)	0.74 ± 0.01
9	Docosanoic acid (Behenic Acid)	0.69 ± 0.02
10	Tetradecanoic acid (Myristic acid)	0.42 ± 0.13
